# Development and Optimization of an Apremilast-Loaded Nanoemulsion Gel for Topical Psoriasis Treatment with In Vitro Anti-Inflammatory Studies Using RAW 264.7 Cells

**DOI:** 10.3390/ph19050691

**Published:** 2026-04-28

**Authors:** Mustafa Saleem Sawan, Mohammad Shah Faisal, Nagia Ahmed El-Megrab, Hanan Mohammed El-Nahas

**Affiliations:** 1 Department of Pharmaceutics and Industrial Pharmacy, Faculty of Pharmacy, Zagazig University, Zagazig 44519, Egypt; nagia.megrab@yahoo.com (N.A.E.-M.); hananelnahas@gmail.com (H.M.E.-N.); 2 Department of Pharmaceutics and Industrial Pharmacy, Faculty of Pharmacy, Elmegrib University, Al-Khoms 218053, Libya; 3 Department of Pharmaceutics and Industrial Pharmacy, Faculty of Pharmacy, Alasmarya Islamic University, Zliten 218521, Libya; m.faisal@asmarya.edu.ly

**Keywords:** psoriasis, apremilast, nanoemulsion gel, skin deposition, anti-inflammatory, RAW 264.7 cell line

## Abstract

**Background:** Apremilast (APM) is a selective phosphodiestrase-4 (PDE-4) inhibitor currently administered orally for the treatment of psoriasis. However, gastrointestinal irritation, frequent dosage regimens, and patient noncompliance limit its oral administration. Additionally, the poor permeability and solubility of APM make dermal administration challenging. **Objective:** The current study aims to formulate an optimized APM-loaded nanoemulsion formulation (APM-NE) to enhance drug delivery to deep psoriatic skin layers, thereby increasing dermal drug concentration for the effective treatment of psoriasis. **Method:** Using the phase titration method, the nanoemulsion (NE) was made with Capryol 90, Tween 20, and Labrasol as oil, surfactant, and co-surfactant, respectively. **Results:** The optimized formulation (F5) exhibited favorable physicochemical properties: mean droplet size of 147.4 ± 2.4 nm, and an entrapment efficiency (EE) reaching 86.30 ± 2.54%. TEM confirmed spherical, uniformly distributed droplets. In vitro release (86.1 ± 0.24%) followed zero-order kinetics. To enhance skin retention, F5 was incorporated into 2% Carbopol 980 gel, yielding F5G with pseudoplastic flow. Ex vivo permeation showed significantly higher drug delivery for F5 (1266.50 ± 5.6 µg/cm^2^) and F5G (1057.7 ± 6.76 µg/cm^2^) compared to crude APM gel (CR-APMG). In vivo, the inhibition of edema in rat paws was highest with F5G (66.83 ± 0.23%). RAW 264.7 cell studies showed 92.37% nitric oxide inhibition, and histopathology confirmed reduced inflammation. **Conclusions:** These results support APM-NE gel as a promising topical strategy for psoriasis therapy.

## 1. Introduction

Psoriasis is a chronic, immune-mediated inflammatory skin disease characterized by abnormal keratinocyte proliferation and persistent inflammation. It affects approximately 2% of the global population, with prevalence rates reaching up to 4.6% in developed countries [1]. Clinically, psoriasis presents as sharply demarcated, erythematous plaques covered with silvery-white scales, resulting from accelerated epidermal turnover and defective keratinocyte differentiation. The pathogenesis of psoriasis is intricately linked to immune system dysfunction, particularly involving aberrant signaling cascades mediated by interleukin-23 (IL-23), growth factors, and adhesion molecules [2]. This immunological dysregulation induces a pro-inflammatory microenvironment, perpetuating the chronic nature of the disease. The disease exhibits considerable heterogeneity, with phenotypes including chronic plaque, guttate, pustular, erythrodermic, scalp, nail, facial, and inverse variants. Psoriatic arthritis further highlights its systemic nature and impact on patient quality of life [3]. Disease severity ranges from isolated, localized plaques to extensive, body-wide involvement, often accompanied by pruritus, erythema, and desquamation [4].

Current therapeutic options depend on disease severity and include topical agents such as corticosteroids [5], vitamin D analogues [6,7], calcineurin inhibitors [8], retinoids [9], coal tar, and anthralin [10,11], for mild cases, while systemic immunosuppressants (methotrexate, cyclosporine, acitretin) and phototherapy are employed in moderate to severe disease [12,13,14,15,16]. Although effective, these treatments are limited by cumulative toxicity, adverse effects, and lack of curative potential. APM, a selective phosphodiesterase-4 (PDE4) inhibitor, represents a more recent oral therapy [17]. By elevating intracellular levels of cyclic adenosine monophosphate (cAMP), APM modulates transcriptional regulators such as transcription factor-kappa B (NF-κB), suppresses pro-inflammatory cytokines (IL-23, tumor necrosis factor-α [TNF-α], interferon-γ [IFN-γ]), and enhances anti-inflammatory mediators [18,19]. Despite its clinical approval for psoriatic arthritis, plaque psoriasis, and Behçet’s disease, oral administration of APM is frequently associated with gastrointestinal disturbances and respiratory tract infections, which compromise patient adherence [20]. APM has demonstrated the ability to suppress nitric oxide synthase activity [21], thereby reducing nitric oxide production, a key mediator in inflammatory pathways. This inhibition curtails the migration of myeloid dendritic cells and macrophages into psoriatic lesions, underscoring the therapeutic potential of APM in topical formulations. To address these limitations, several colloidal carriers of APM have been investigated, including liposomes [22], nanocrystals [23], solid dispersions [24,25], ethosomes [26], transethosomes [27], solid lipid nanoparticles [28], and nanoemulsions [20,29]. These systems primarily aim to improve oral bioavailability through solubilization or encapsulation. However, attempts to develop topical formulations of APM remain scarce. This represents a significant research gap, as topical delivery offers distinct advantages in dermatology, site-specific drug deposition, reduced systemic exposure, and improved patient compliance [30].

Among colloidal systems, NEs are particularly promising for topical application due to their small droplet size, thermodynamic stability, high drug-loading efficiency, and ability to enhance skin permeation [31,32]. However, their inherently low viscosity can compromise retention at the application site and hinder ease-of-use. To address this limitation, NE_S_ are often transformed into gel-based systems to improve topical adherence and patient convenience [33,34].

In light of these considerations, the present study introduces a novel NE-based gel formulation of APM for topical management of psoriasis. This approach is designed to combine the permeation-enhancing properties of NE_S_ with the practicality of gels, thereby achieving localized anti-inflammatory effects while minimizing systemic side effects. The formulation was prepared, characterized, and evaluated in vitro using RAW 264.7 murine macrophages and in vivo models, providing comprehensive insights into its therapeutic potential for psoriatic inflammation.

## 2. Results and Discussion

### 2.1. Screening the Solubility of APM in Various Oils, Surfactants, and Co-Surfactants

In this study, various types of oils such as natural, semi-synthetic, and synthetic were used to determine the solubility of APM. The selection of the suitable oil phase is crucial, as it significantly influences drug loading and solubilization efficiency in NE systems [35]. Among the tested oils, Capryol 90 (HLB = 5) demonstrated the highest solubility for APM (2.62 ± 0.07 mg/mL) and was therefore identified as the optimal oil phase.

For identifying the most appropriate surfactant and co-surfactant, both solubility potential and hydrophilic-lipophilic balance (HLB) values were considered. Among surfactants, Tween 20 exhibited the maximum solubility (1.98 ± 0.03 mg/mL), while Labrasol showed superior solubility among co-surfactants (2.63 ± 0.06 mg/mL). An effective surfactant should reduce interfacial tension, form a stable and flexible interfacial film, and possess an appropriate HLB value to facilitate the desired curvature for oil-in-water (*o*/*w*) NE formation. Since achieving optimal interfacial properties with a single surfactant is often insufficient, the inclusion of a co-surfactant is necessary to stabilize the system and transiently reduce interfacial tension [34].

Literature suggests that a combined HLB value exceeding 10 is essential for efficient emulsification and the formation of thermodynamically stable *o*/*w* NE_S_ [36]. Accordingly, Tween 20 (HLB = 16.7), a non-ionic and biocompatible surfactant, was selected alongside Labrasol (HLB = 12) as the co-surfactant to support the formulation of a stable NE system.

### 2.2. Development of Pseudo-Ternary Phase Diagrams

**Figure 1:**
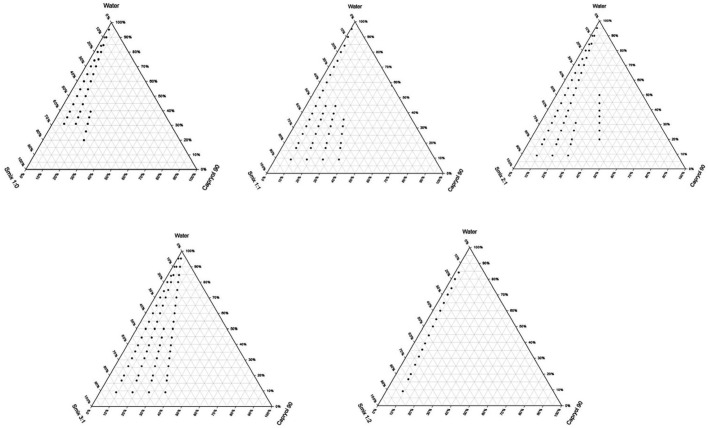
Pseudo-ternary phase diagrams constructed at different surfactant/co-surfactant (Smix) ratios, illustrating nanoemulsion formation regions.

To identify the optimal NE region, Pseudo-ternary phase diagrams were constructed utilizing Capryol 90 as the oil phase, a surfactant mixture (Smix) comprising Tween 20 and Labrasol, and distilled water as the aqueous phase (Figure 1). The ratio of surfactant to co-surfactant (Smix) was found to significantly influence the extent of the NE region within the diagram. An expansion of the NE zone was observed with increasing Smix ratios, following the trend: 1:1 < 2:1 < 3:1. This indicates that higher surfactant proportions enhance emulsification efficiency and facilitate the formation of thermodynamically stable NE_S_. Although increasing the surfactant-to-co-surfactant ratio to 4:1 resulted in the disappearance of the NE region, ratios between 1:1 and 3:1 demonstrated a progressive expansion of the NE zone. This enhancement is attributed to the reduction in interfacial tension, leading to smaller droplet sizes and increased surface area, thereby promoting the formation of thermodynamically stable NE_S_. Conversely, elevating the co-surfactant proportion in Smix from 1:1 to 1:3 caused a contraction of the NE region (Figure 1) and was associated with increased instability during storage, likely due to the disruptive effect of excessive co-surfactant on interfacial film integrity [34]. To further evaluate formulation robustness, selected NE_S_ underwent a series of stress tests, including centrifugation, freeze–thaw cycles, and thermal cycling. Formulations were chosen based on their position within the pseudo-ternary phase diagram for each Smix ratio.

The criteria for selecting the optimized formulation included maximizing oil content to enhance APM solubilization, while ensuring the total surfactant concentration remained below 60% to maintain suitability for topical application [37].

### 2.3. Thermodynamic Stability Study

**Figure 2:**
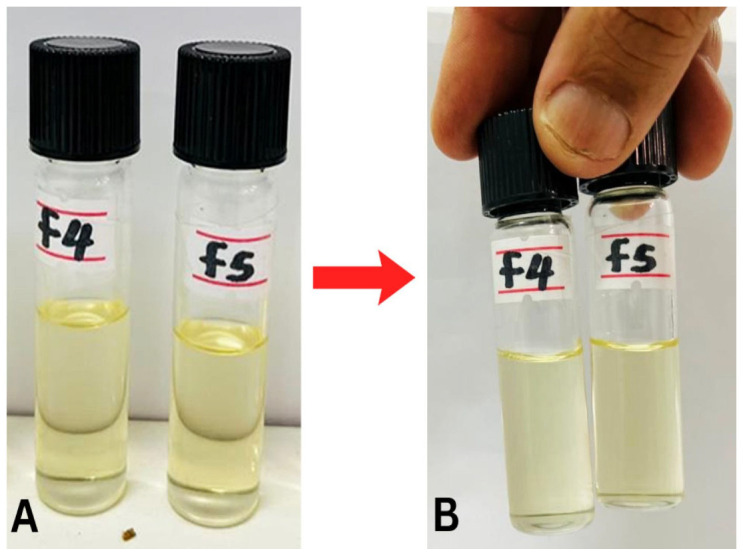
Photographic images of nanoemulsion (NE) formulations before (**A**) and after (**B**) thermodynamic stability studies.

Generally, NE_S_ exhibit kinetic stability because of their small droplet size and appropriate surfactants and provide resistance against droplet coalescence and phase separation over an extended period of storage time [33]. To ensure the elimination of metastable or unstable formulations, thermodynamic stability testing was performed on all optimized APM-NE_S_ (F1–F6). Each formulation was subjected to centrifugation, freeze–thaw cycles, and thermal stress through heating and cooling cycles. No evidence of destabilizing mechanisms including flocculation, sedimentation, coalescence, or phase separation was observed, confirming their thermodynamic robustness as shown in Figure 2.

Additionally, formulation composition was found to influence droplet size distribution. Increased oil content was associated with smaller particle sizes, likely due to enhanced solubilization and interfacial stabilization, whereas higher water content tended to produce larger droplets [38].

### 2.4. Characterization of Optimized Formulation

#### 2.4.1. Physicochemical Characteristics of APM-Loaded NE Formulations

The APM-NE formulations (F1–F6) were evaluated for mean globule size, polydispersity index (PDI), and zeta potential. The mean globule size ranged from 147.4 ± 2.4 to 275.5 ± 8.2 nm, with all formulations falling below 300 nm, confirming their nanoscale nature and suitability for enhanced dermal penetration and thermodynamic stability. PDI values varied between 0.447 ± 0.008 and 0.623 ± 0.005, indicating adequate homogeneity of droplet distribution, as greater polydispersity index values indicate a weaker uniformity of droplet size in the formulations [39]. The values of zeta potential ranged from −2.87 ± 0.21 to −2.03 ± 0.18 mV, reflecting low surface charge yet sufficient stability under stress conditions.

Formulation F5 exhibited the most favorable characteristics, including the smallest droplet size, lowest PDI, and acceptable zeta potential as presented in Table 1. Despite the low zeta potential, all formulations remained stable during thermodynamic testing, suggesting that the electrostatic stabilization is not the main mechanism responsible for the stability of NE_S_ with low zeta potential values. Instead, their stability is achieved through steric repulsion. This effect is attributed to the use of nonionic surfactants such as Tween 20, which form a compacted steric layer around the globules, preventing aggregation [35,40].

Additionally, refractive index (RI) and transmittance (%T) values were assessed to evaluate optical clarity. RI values ranged from 1.338 ± 0.04 to 1.361 ± 0.02, while %T values ranged from 96.94 ± 0.48 to 99.57 ± 0.33%. Formulation F5 demonstrated the lowest RI (1.338 ± 0.04) and highest transmittance (99.57 ± 0.33%), indicating excellent emulsification and transparency, characteristic of isotropic NE systems [20,41].

Transmission electron microscopy (TEM) analysis of all formulations confirmed the presence of spherical globules with a uniform distribution, and the observed sizes consistently remained below 100 nm. Notably, these values were smaller than those obtained using the Malvern particle size analyzer. This discrepancy can be attributed to methodological differences: during TEM grid preparation, air drying may induce partial shrinkage or deformation of droplets, thereby reducing their apparent size compared to their hydrated state [42]. Furthermore, the measurement principles of the two techniques differ substantially. TEM provides number-weighted size distributions, whereas dynamic light scattering (DLS) reports intensity-weighted hydrodynamic diameters, which are disproportionately influenced by the presence of larger particles within the population [43].

**Figure 3:**
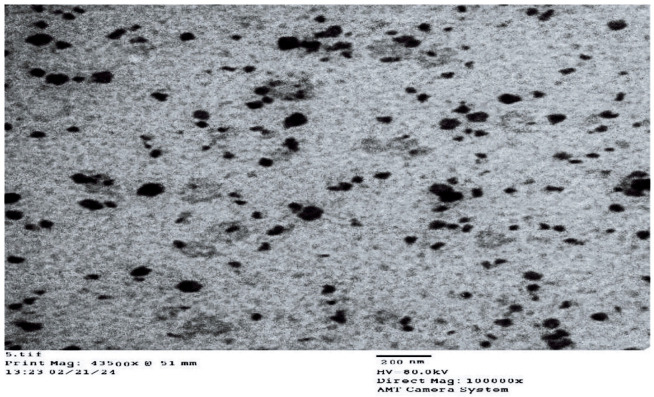
Transmission electron microscopy (TEM) image of the optimized nanoemulsion (F5).

Transmission electron microscopy (TEM) images of all formulations confirmed the spherical shape and uniform distribution of globules, with globule sizes consistently below 100 nm across all formulations (Figure 3).

#### 2.4.2. Viscosity and pH

The viscosity of APM-NE formulations ranged from 0.044 ± 0.002 to 0.056 ± 0.007 Pa·s. A linear correlation between shear rate and shear stress was observed, confirming Newtonian flow behavior, wherein viscosity remained constant across varying shear rates. An increase in Tween 20 concentration led to elevated viscosity values, attributable to the inherently higher apparent viscosity of Tween 20 (0.250–0.450 Pa·s). As expected, the low viscosity of the NE formulations aligns with previous findings [44], but may pose challenges in topical application due to limited spreadability and reduced retention time on the skin surface. To overcome these limitations, conversion of NEs into nanogel systems is recommended to enhance skin adherence and prolong drug residence time.

The pH of topical preparations is correlated with both skin irritation and optimizing drug delivery [45]. The mean pH values of the NE formulations were found to be within the acceptable range for dermal application (5.93 ± 0.04 to 6.59 ± 0.02), indicating their suitability for topical administration without risk of irritation or disruption to the skin’s natural barrier function.

#### 2.4.3. Entrapment Efficiency

The efficacy of a nanocarrier in retaining the drug and delivering the product to the intended location is determined by its entrapment efficiency (EE) [46]. All APM-NE formulations demonstrated high entrapment efficiencies, ranging from 78.97 ± 2.12% to 86.30 ± 2.54%. Among these, formulation F5 exhibited the highest %EE, indicating superior drug retention compared to other formulations.

The enhanced entrapment observed in F5 is attributed to the solubility of APM in Capryol 90 and its compatibility with other ingredients. Given the hydrophobic nature of APM, the drug preferentially partitions into the oil droplets, which are further stabilized by the interfacial film formed by Tween 20 and Labrasol. This structural arrangement effectively prevents drug leakage and contributes to the overall stability of the NE [47].

### 2.5. Characterization of Drug–Excipient Compatibility

**Figure 4:**
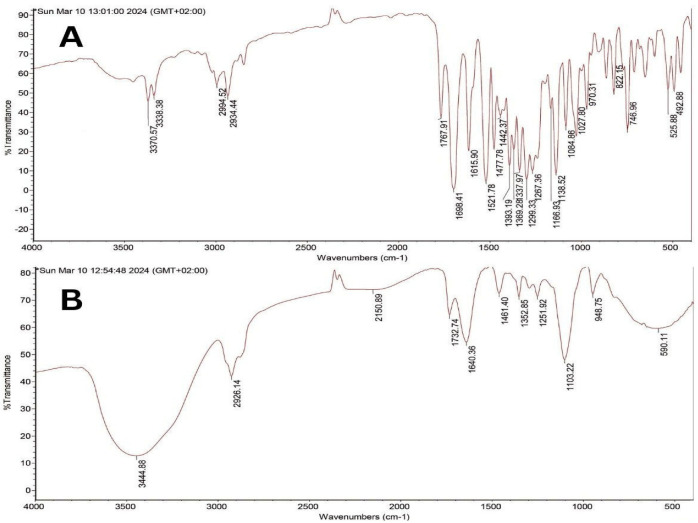
FTIR spectra of APM (**A**) and APM-loaded NE (F5) (**B**), showing retention of characteristic APM peaks within the formulation. Abbreviations: FTIR, Fourier transform infrared spectroscopy; APM, apremilast; NE, nanoemulsion; F5, optimized apremilast nanoemulsion formulation.

The FTIR spectrum of pure APM exhibited characteristic peaks corresponding to its major functional groups, including N–H stretching (3370 cm^−1^), C–H stretching (2930–2990 cm^−1^), and C=O stretching (1768 cm^−1^), consistent with previously reported data [48] (Figure 4A). In the optimized NE formulation (F5), these key peaks were retained (N–H stretching at 3445 cm^−1^, C–H stretching at 2923 cm^−1^, and C=O stretching at 1733 cm^−1^), with a reduction in intensity indicating drug entrapment within the oil globules (Figure 4B). The preservation of these characteristic bands without significant shifts or disappearance confirms the absence of chemical incompatibility between APM and the excipients, supporting the stability of the formulation.

### 2.6. In Vitro Drug Release and Release Kinetics

**Figure 5:**
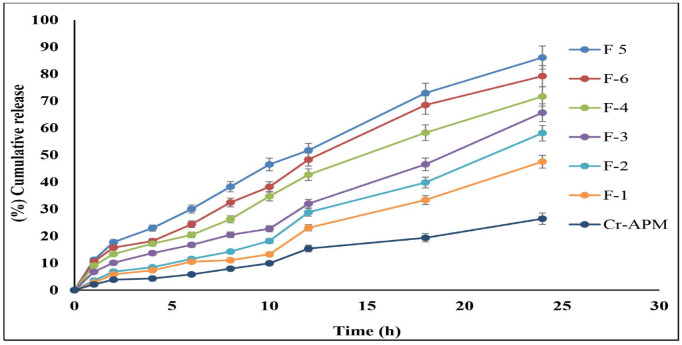
In vitro release of APM from NE formulations (F1–F6) compared with CR-APM dispersion. Results are expressed as mean ± standard deviation (*n* = 3). Abbreviations: APM, apremilast; NE, nanoemulsion; CR-APM, crude apremilast dispersion.

In vitro drug release studies were conducted to evaluate the release behavior of APM from NE formulations and to elucidate the underlying release mechanisms. These studies provide critical insights into the kinetics of drug liberation, thereby supporting the rational design and optimization of topical delivery systems. The cumulative drug release over 24 h for the APM-NE formulations was as follows: F5 (86.1 ± 0.24%), F6 (79.2 ± 0.21%), F4 (71.6 ± 0.37%), F3 (65.7 ± 0.18%), F2 (58.1 ± 0.25%), and F1 (47.52 ± 0.31%). In contrast, the CR-APM dispersion exhibited a lower release (26.4 ± 0.39%), underscoring the superior performance of the NE systems (Figure 5).

The enhanced release from NE formulations is attributed to their nanoscale droplet size, which provides a larger surface area available for drug diffusion and is attributed to increased drug solubilization capacity, low viscosity, and the presence of surfactants or co-surfactants, which are recognized factors that contribute to improved drug release [35,38]. These factors collectively contribute to improved drug availability in the aqueous phase, thereby enhancing release kinetics [20].

To elucidate the release mechanism, the experimental data were analyzed using various kinetic models, including zero-order, first-order, and Higuchi equations [17]. Among these, the zero-order model exhibited the strongest coefficient of determination (R^2^ = 0.9803), indicating that APM release from the NE formulations followed a constant, concentration-independent rate. This controlled release pattern enables uniform delivery of APM over time, thereby maintaining stable drug levels within the skin. Such a release pattern is particularly advantageous in psoriasis therapy, as it minimizes fluctuations in drug concentration that may otherwise lead to irritation or reduced therapeutic efficacy. Sustained and predictable release is therefore critical for achieving consistent anti-inflammatory effects in chronic conditions like psoriasis. The Higuchi model also demonstrated a good fit (R^2^ = 0.9671), suggesting that diffusion plays a contributory role in the release process. In contrast, the first-order model showed a relatively weak coefficient of determination (R^2^ = 0.5754), indicating that the release was not predominantly concentration-dependent. Collectively, these findings suggest that the NEs primarily follow zero-order kinetics, with secondary diffusion-driven release, and negligible alignment with first-order kinetics.

### 2.7. Evaluation of APM-NEG

#### 2.7.1. Visual Characterization of APM-NEG

The formulated APM-NE gel was transparent and glossy in appearance. There was no sign of phase separation.

#### 2.7.2. pH and Viscosity

The pH values of the optimized NEG (F5G) and CR-APMG were found to be 6.3 ± 0.03 and 6.7 ± 0.04, respectively (Table 2). These values fall within the adequate range for topical formulations, indicating minimal risk of skin irritation and compatibility with dermal tissues [17].

Viscosity measurements for both gel formulations were conducted in triplicate, and the mean viscosity values are demonstrated in Table 2. 

**Figure 6:**
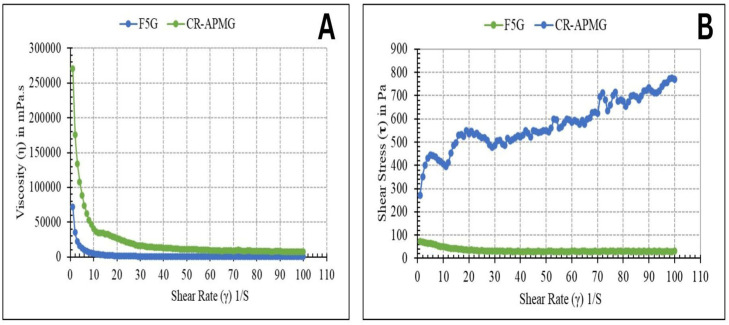
Rheological behavior of F5G and CR-APMG. (**A**) Viscosity (η) as a function of shear rate (γ). Both formulations exhibit a decrease in viscosity with increasing shear rate, indicating shear-thinning behavior. (**B**) Shear stress (τ) as a function of shear rate (γ). F5G shows a nonlinear increase in shear stress with increasing shear rate, confirming non-Newtonian pseudoplastic flow, whereas CR-APMG displays lower shear stress values. Abbreviations: F5G, Optimized apremilast nanoemulsion gel; CR-APMG, Crude apremilast gel.

The rheological profiles as illustrated in Figure 6A,B confirm that both formulations possess suitable viscosity for topical administration, ensuring adequate spreadability and retention on the skin surface.

Viscosity acts as a pivotal factor in the design of topical drug therapy, influencing formulation stability, spreadability, drug release kinetics, and overall application performance [49]. The rheological properties of topical gels are largely governed by the type and concentration of polymers and excipients incorporated. As presented in Table 2, a statistically very highly significant difference (*p* < 0.001) was found between the viscosities of the NE (F5), NEG (F5G), and CR-APMG.

The reduced viscosity of F5G is due to the inclusion of low-viscosity components such as Capryol 90 (oil phase), Tween 20 (surfactant), and Labrasol (co-surfactant), which collectively exhibit non-Newtonian pseudoplastic flow behavior (Figure 6A). In contrast, the higher viscosity of the crude gel formulation is due to the dispersion of APM within a Carbopol 980 polymer matrix, which imparts a more structured and cohesive gel network. This increased viscosity is advantageous for topical application, as it enhances skin coverage and prolongs residence time at the site of application [50].

#### 2.7.3. Spreadability

Spreadability is a key parameter in the development of topical gel formulations, reflecting their ability to uniformly distribute across the surface of the skin. A larger spread diameter corresponds to greater coverage, which enhances therapeutic efficacy and improves patient compliance [51]. Spreadability is inversely related to viscosity; formulations with lower viscosity typically exhibit better spreadability.

In this study, the APM-NEG (F5G) and CR-APMG demonstrated spreadability diameters of 6.5 cm and 4.1 cm, respectively, corresponding to spreadability percentages of 650% and 410% (Table 2). These results indicate a statistically very highly significant difference between F5G and CR-APMG formulations (*p* < 0.001), with F5G exhibiting superior spreadability. The enhanced performance of F5G is attributed to its lower viscosity, resulting from the use of low-viscosity excipients such as Capryol 90, Tween 20, and Labrasol. In contrast, the reduced spreadability of CR-APMG is linked to its higher viscosity resulting from the incorporation of APM into a Carbopol 980 polymer matrix. The spreadability of F5G falls within the acceptable range (5–7 cm), confirming its suitability for topical application.

### 2.8. Drug Content

The drug content of the optimized NE (F5), NEG formulation (F5G), and CR-APMG was quantitatively assessed to evaluate uniformity and mixing efficiency. The measured drug content values were 99.15% for F5, 97.82% for F5G, and 96.82% for CR-APMG, all of which fall within pharmaceutically acceptable limits (Table 2). These results confirm the homogenous distribution of APM throughout each formulation, indicating effective incorporation and consistent drug loading across the gel matrix.

### 2.9. Ex Vivo Skin Permeation and Flux Profile Studies

**Figure 7:**
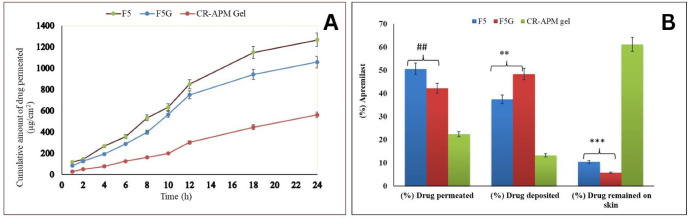
(**A**) Ex vivo skin permeation profiles of APM from NE (F5), NE gel (F5G), and CR-APM gel. (**B**) Ex vivo drug permeation, deposition, and retention of APM in rat skin from F5 and F5G compared with CR-APM gel. Results expressed as mean ± standard deviation (*n* = 3). Statistical differences were assessed by one-way ANOVA with Tukey’s post hoc test; ## *p* < 0.05, ** *p* < 0.01, *** *p* < 0.001 vs. CR-APM gel. Abbreviations: F5, optimized apremilast nanoemulsion formulation; F5G: optimized apremilast nanoemulsion gel; CR-APMG, crude apremilast gel; ANOVA, analysis of variance.

The skin permeation potential of APM from various formulations was evaluated using rat skin over a period of 24 h. The optimized NE (F5) demonstrated the highest cumulative drug permeation (1266.50 ± 5.6 µg/cm^2^), followed by the NEG formulation (F5G) with 1057.7 ± 6.76 µg/cm^2^. In contrast, the CR-APMG exhibited significantly lower permeation (561.38 ± 3.21 µg/cm^2^) (Figure 7A). These results indicate a 2.02-fold and 1.70-fold enhancement in drug permeation for F5 and F5G, respectively, compared to CR-APMG, highlighting the superior solubility and permeability of APM in the NE-based systems.

The flux values for F5 and F5G were 28.90 ± 0.68 µg/cm^2^/h and 24.31 ± 0.19 µg/cm^2^/h, respectively, both markedly higher than that of CR-APMG (14.28 ± 0.15 µg/cm^2^/h). Correspondingly, the permeability coefficients were 0.0145 ± 0.002 cm^2^/h for F5, 0.0097 ± 0.001 cm^2^/h for F5G, and 0.0057 ± 0.01 cm^2^/h for CR-APMG. Enhancement ratios calculated for F5 and F5G were 2.024 and 1.702, respectively (Table 3).

The enhanced permeation observed in F5 is attributed to its nanoscale globules size, which provides a large surface area and facilitates drug diffusion across the stratum corneum. In contrast, the gel matrix in F5G, formed by a three-dimensional hydrogel network, slows drug release and slightly reduces permeation efficiency. Nonetheless, both formulations significantly (*p* < 0.05) outperformed the CR-APMG, confirming the suitability of NE-based systems for enhanced topical delivery of APM.

### 2.10. Ex Vivo Skin Deposition Studies

The amount of APM retained within the skin tissue following topical application was quantified to assess the deposition efficiency of different formulations (Figure 7B). The optimized NE (F5) and NEG (F5G) exhibited significantly (*p* < 0.01) higher drug deposition percentages of 37.5 ± 1.01% and 48.4 ± 1.38%, respectively. In contrast, the CR-APMG demonstrated a markedly lower deposition of 13.30 ± 1.04%, with very low permeation through the skin. This limited retention is attributed to the poor solubility and low penetration capacity of APM when dispersed in a Carbopol-based gel matrix.

For effective management of psoriasis, it is crucial to employ topical formulations that promote enhanced drug accumulation within the epidermal layer while minimizing transdermal permeation. Such deposition-focused delivery ensures sustained therapeutic action at the target site and reduces systemic exposure [52].

The superior deposition observed in F5G highlights the potential of NEG systems to improve localized drug delivery and therapeutic outcomes in dermatological applications. The CR-APMG exhibited a very highly significant amount of APM (85.26 ± 6.5%) leftover on the skin tissue (*p* < 0.001) compared with the optimized NE (F5) and NEG (F5G), which is due to the low solubility and permeability of CR-APMG.

### 2.11. In Vivo Anti-Inflammatory Study

**Figure 8:**
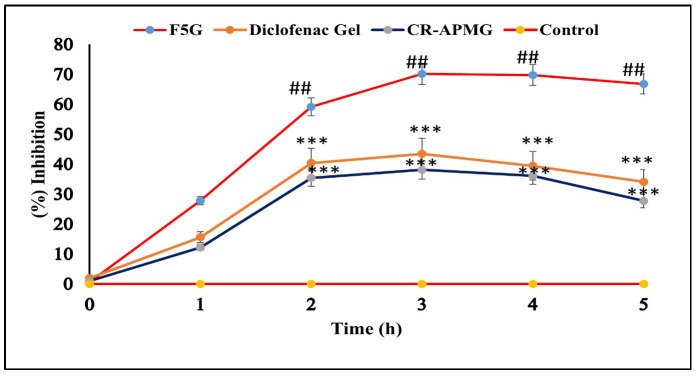
Percentage inhibition of carrageenan-induced paw edema in rats. F5G gel showed significant inhibition versus control (## *p* < 0.01) and marketed diclofenac gel (*** *p* < 0.01), while APM gel exhibited no significant difference compared to diclofenac gel (*p* > 0.05). Statistical differences among groups were analyzed by one-way ANOVA followed by Tukey’s post hoc test. Abbreviations: F5G, optimized apremilast nanoemulsion gel; CR-APMG, crude apremilast gel; ANOVA, analysis of variance.

The anti-inflammatory effectiveness of the optimized NEG formulation (F5G), CR-APMG, and a marketed 1% diclofenac sodium gel was assessed using the rat paw edema model (Figure 8). The F5G formulation demonstrated significant inhibition of paw edema at all-time points, with values of 27.89 ± 0.98%, 59.22 ± 0.72%, 70.23 ± 0.82%, 69.75 ± 0.31%, and 66.83 ± 0.23% at time points of 1, 2, 3, 4, and 5 h, respectively. In comparison, diclofenac sodium gel showed lower inhibition rates of 15.64 ± 0.35%, 40.42 ± 0.61%, 43.51 ± 0.84%, 39.49 ± 0.28%, and 34.15 ± 0.72%, while CR-APMG exhibited 12.24 ± 0.69%, 35.46 ± 1.36%, 38.17 ± 0.55%, 36.13 ± 0.84%, and 27.80 ± 0.91% inhibition over the same time intervals. Maximum edema inhibition was observed at the 3-h mark across all formulations. The superior performance of F5G is linked to its nanoscale globules, which improve skin penetration and facilitate efficient drug delivery to the inflamed tissue. Statistical analysis confirmed that the anti-inflammatory effect of F5G was significantly greater (*p* < 0.01) than that of both CR-APMG and diclofenac sodium gel at 2, 3, 4, and 5 h. However, no significant difference (*p* > 0.05) was found between APM gel and diclofenac gel. These findings underscore the potential of NEG systems in improving topical anti-inflammatory therapy.

### 2.12. Histopathological Study

**Figure 9:**
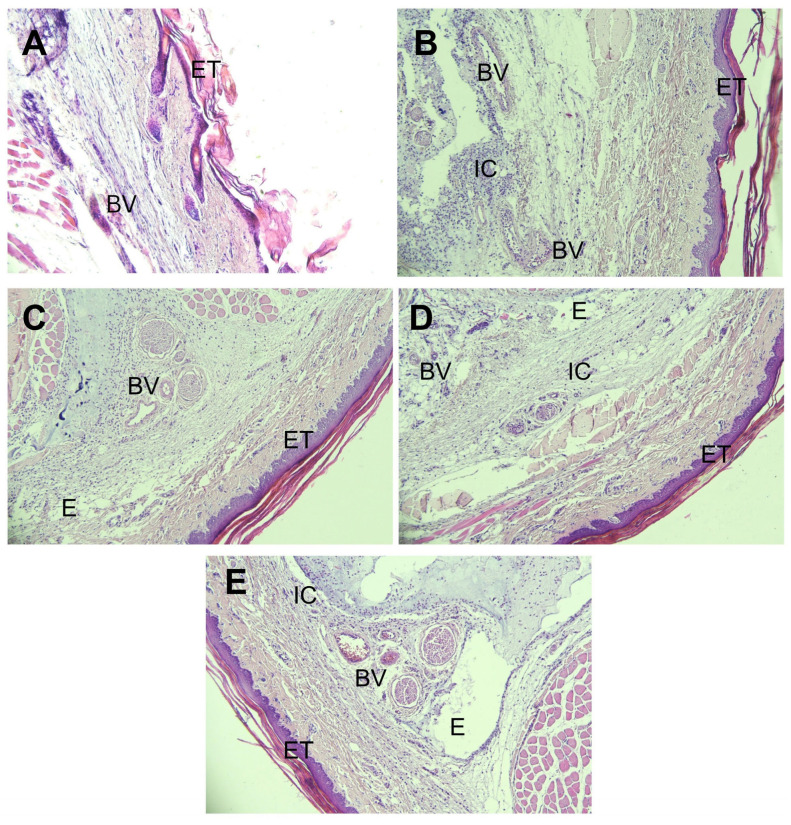
Representative photomicrographs of rat hind paws (100× magnification): (**A**) negative control; (**B**) placebo nanoemulsion gel; (**C**) optimized apremilast nanoemulsion gel; (**D**) marketed diclofenac emulgel; (**E**) crude apremilast gel. Abbreviations: E, edema; BV, blood vessels; IC, inflammatory cells; ET, epidermal thickness. Scale bar = 200 µm.

Figure 9A represents the negative control group (untreated), which was not injected with carrageenan and shows normal skin architecture. In contrast, following sub plantar injection of 0.1% carrageenan in other experimental groups, Group I (placebo gel) exhibited pronounced inflammatory responses, including extensive infiltration of mononuclear inflammatory cells (IC), marked edema (E), increased epidermal thickness (ET), and severe vascular congestion and dilation (BV) (Figure 9B), confirming the absence of anti-inflammatory activity in the placebo formulation.

Graph 5G demonstrated substantial attenuation of inflammatory markers, including reduced edema, normalized dermal thickness, diminished vascular congestion, and minimal inflammatory cell infiltration (Figure 9C). These effects were notably superior to those observed in Group III (diclofenac sodium gel; Figure 9D) and Group IV (CR-APMG; Figure 9E).

The significant histological improvements in Group II are attributed to the enhanced anti-inflammatory efficacy of the NE-based gel, likely mediated by its ability to suppress pro-inflammatory cytokine production and improve drug penetration due to its nanoscale droplet architecture [53]. These results highlight the therapeutic potential of APM-NE gel as an effective topical intervention for psoriasis and other inflammatory skin disorders.

### 2.13. Microphage Cell Line Anti-Inflammatory Studies

#### 2.13.1. Cell Viability

**Figure 10:**
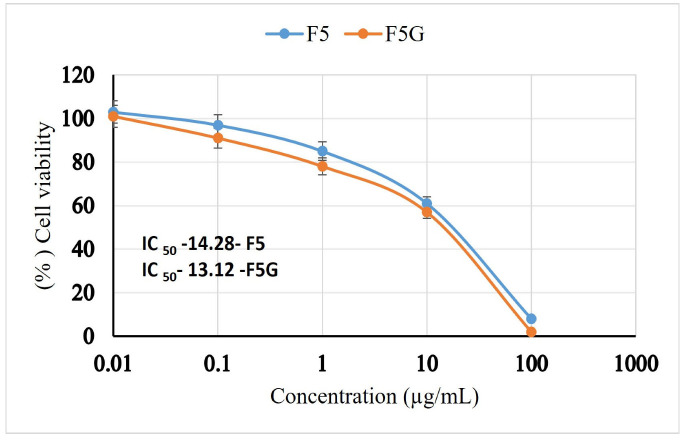
Effect of apremilast nanoemulsion and apremilast nanoemulsion gel on RAW 264.7 cell viability. Values are expressed as mean ± standard deviation (*n* = 3). Abbreviations: IC_50_, represents the half-maximal inhibitory concentration.

**Figure 11:**
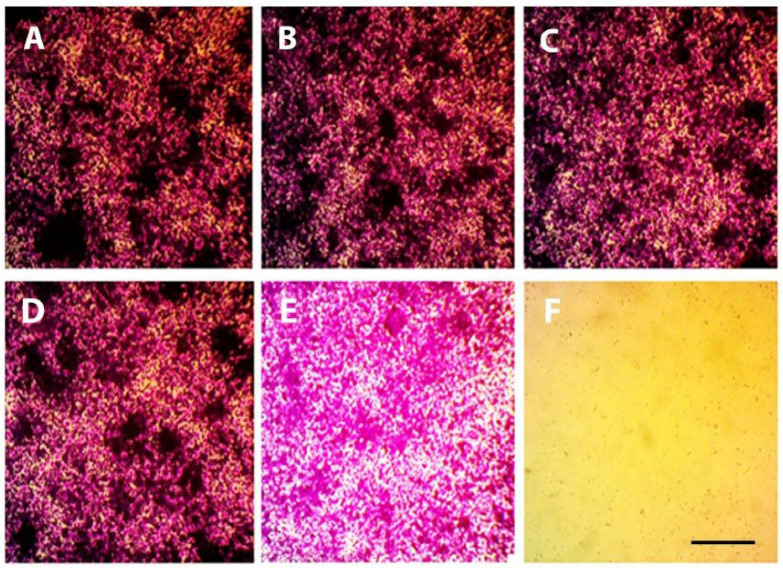
Optical microscope images of RAW 264.7 macrophages from the cell viability assay (100× magnification): (**A**) quercetin control; (**B**) NEG 0.01 µg/mL; (**C**) NEG 0.1 µg/mL; (**D**) NEG 1.0 µg/mL; (**E**) NEG 10.0 µg/mL; and (**F**) NEG 100.0 µg/mL. Scale bar = 100 µm. Abbreviations: NEG, apremilast nanoemulsion gel; RAW 264.7, murine macrophage cell line.

The cytotoxic potential of APM-loaded NE (APM-NE) and NEG (APM-NEG) formulations was evaluated using the Sulforhodamine B (SRB) assay on RAW 264.7 murine macrophage cells cultured in 96-well plates. The IC_50_ values for APM-NE and APM-NEG were 14.28 µg/mL and 13.12 µg/mL, respectively, demonstrating dose-dependent cytotoxicity (Figure 10 and Figure 11). The reduction in cell viability observed at higher concentrations is likely due to the presence of Tween 20, a surfactant known to exert cytotoxic effects at higher concentrations [54]. Additionally, the slightly greater cytotoxicity of APM-NEG may be linked to the inclusion of triethanolamine, used as a neutralizing agent in the Carbopol gel matrix, which has also been reported to affect cell viability [55].

Importantly, this cytotoxicity is not detrimental in the context of psoriasis treatment. On the contrary, it may contribute to therapeutic efficacy by promoting apoptosis in hyper proliferative keratinocytes, a hallmark of psoriatic lesions. Psoriasis is characterized by disrupted epidermal homeostasis, where reduced keratinocyte apoptosis leads to excessive proliferation and release of pro-inflammatory cytokines [56,57]. By inducing apoptosis, both Tween 20 and triethanolamine may help restore the balance between cell growth and death, thereby attenuating inflammation and hyper proliferation.

These findings suggest that APM-NEG not only delivers APM effectively, but also enhances its therapeutic action through synergistic cytotoxic mechanisms, positioning it as a promising topical candidate for psoriasis management.

#### 2.13.2. Anti-Inflammatory Assay

Nitric oxide (NO) production is a key indicator of inflammatory response in RAW 264.7 macrophages. Upon stimulation with lipopolysaccharide (LPS, 1 µg/mL), NO levels in the culture supernatant increased significantly, reaching approximately 50 µM. To assess the anti-inflammatory activity of APM, NO levels were quantified using the Griess reagent in both untreated and APM-treated cells.

Two concentrations of APM 0.01 µg/mL and 0.1 µg/mL were assessed in NE and NEG formulations, with quercetin (30 µM) serving as a positive control. APM-NE reduced NO production by 23.42% and 87.27%, while APM-NEG achieved reductions of 25.58% and 92.37%, respectively, compared to the LPS-treated group after 48 h. The most pronounced inhibition was observed with APM-NEG at 0.1 µg/mL, highlighting its strong anti-inflammatory activity.

These findings indicate that both APM-NE and APM-NEG effectively suppress LPS-induced NO production, with the NEG showing slightly higher inhibition. The results underscore the therapeutic potential of APM-loaded nanoformulations in managing inflammatory conditions such as psoriasis, where excessive NO production contributes to disease pathology.

## 3. Materials and Method

### 3.1. Materials

APM was procured from Beijing Mesochem Technology Co., Ltd. (Beijing, China). Labrasol and Capryol 90 were kindly provided by Gattefossé Co. (Saint-Priest, France). Tween 20 was obtained from Riedel-De Haën AG (Seelze, Hannover, Germany), while triethanolamine was sourced from Sigma-Aldrich Chemie GmbH (Steinheim, Germany). Carbopol 980 was supplied by Henan Jinhe Co., Ltd., (Zhengzhou, China), and carrageenan was purchased from Almolok Chemicals (Cairo, Egypt). The RAW 264.7 murine macrophage cell line was acquired from Nawah Scientific Inc. (Mokatam, Cairo, Egypt). HPLC-grade acetonitrile and methanol were sourced from Sigma-Aldrich (St. Louis, MO, USA). Milli-Q water, generated in-house using a Milli-Q purification system (EMD Millipore, Billerica, MA, USA), was used throughout all experimental procedures. All other reagents and chemicals were of analytical grade.

### 3.2. Methods

#### 3.2.1. Solubility Screening of APM in Selected Oils, Surfactants, and Co-Surfactants

To determine the optimal excipients for NE formulation, solubility studies were conducted by incorporating an excess amount of APM into 2 mL of various oils, surfactants, and co-surfactants. The oils evaluated included Capryol 90, Labrafac Lipophile WL 1349, isopropyl myristate (IPM), glycerol trioleate, olive oil, eucalyptus oil, linseed oil, castor oil, and oleic acid. Surfactants tested comprised Plurol Diisostearique, Labrafil M, Tween 20, Tween 60, Tween 80, Tween 85, Span 20, and Span 80. Co-surfactants included Lauroglycol FCC, Labrasol, propylene glycol, polyethylene glycol 400, and Transcutol P. Each mixture was prepared in 5 mL capped vials and homogenized using a vortex mixer (Vortex MS 3 basic, IKA^®^-Werke GmbH & Co., Staufen, Germany). The capped vials were continuously agitated for 72 h at ambient temperature (25 ± 2 °C) using a digital orbital shaker (SHO-2D, WITEG Labortechnik GmbH, Wertheim, Germany). After equilibration, the samples were subjected to centrifugation to separate undissolved APM, followed by filtration. The concentration of solubilized APM was determined using a validated reverse-phase high-performance liquid chromatography (RP-HPLC) method.

#### 3.2.2. Development of Pseudo-Ternary Phase Diagrams

Pseudo-ternary phase diagrams were developed using the phase titration method to determine the NE region within various component ratios. This technique involved the incremental addition of distilled water (ranging from 5% to 95% *v*/*v* in 5% intervals) to mixtures of oil, surfactant, and co-surfactant (S_mix_) at predetermined weight ratios. The resulting mixtures were homogenized using a vortex mixer at ambient temperature (25 ± 2 °C) as described by Spiclin et al. (2003) [58]. S_mix_ ratios evaluated included 1:0, 1:1, 1:2, 1:2.5, 1:3, 2:1, 2.5:1, and 3:1. For each S_mix_ ratio, twelve distinct oil-to-S_mix_ weight ratios were tested: 1:9, 1:8, 1:7, 1:6, 1:5, 1:4, 1:3, 1:2, 1:1, 6:4 (1:0.7), 7:3 (1:0.43), and 9:1. The S_mix_ was titrated with water until a thermodynamically stable, translucent, and isotropic NE was obtained. The corresponding water content was recorded, and the pseudophase diagrams were plotted to define the NE region, representing the proportions of oil, S_mix_, and water employed [59,60].

#### 3.2.3. Thermodynamic Stability Studies

NE formulations were exposed to six consecutive heating (45 °C) and cooling (4 °C) cycles, each lasting 48 h, to evaluate their thermodynamic stability [20]. Post-cycling, the formulations were examined for thermodynamic stability factors, including opalescence, creaming, and phase separation. Formulations that remained physically stable were further evaluated by centrifugation at 3500 rpm for 30 min at ambient temperature (25 ± 2 °C) [61]. Additionally, they were subjected to freezing and thawing cycles at −21 °C and +25 °C, respectively, each for a duration of 48 h [62]. NEs that successfully passed all these stress tests were deemed thermodynamically stable.

#### 3.2.4. Preparation of APM-Loaded-NEs

NE formulations containing APM were developed based on compositions derived from pseudo-ternary phase diagrams (Table 4). A precise concentration of APM (2.5 mg/mL; 0.25% *w*/*v*) was dissolved in Capryol 90 (7–18%) and S_mix_ (33–50%), comprising Tween 20 (surfactant) and Labrasol (co-surfactant), under constant stirring at 700 rpm until complete solubilization occurred. Thereafter, distilled water was incrementally added dropwise under constant agitation until the formation of clear, isotropic, and low-viscosity systems, indicative of NEs, was observed [63]. The resulting formulations were transferred into screw-capped vials and stored at ambient temperature for subsequent monitoring of physical stability.

#### 3.2.5. Characterization of APM-NEs

##### Globule Size, Size Distribution, and Zeta Potential

One mL of each NE formulation was appropriately diluted with deionized water and analyzed for droplet size and size distribution parameters, including mean volume diameter and polydispersity index (PDI), using photon correlation spectroscopy. Measurements were performed using a Zetasizer Nano-ZS (Malvern Panalytical Ltd., Malvern, UK) at a fixed scattering angle of 173° and a controlled temperature of 25 °C. The analysis of the samples was conducted in triplicate to ensure experimental reliability. Zeta potential measurements were carried out using the same device.

##### Transmission Electron Microscopy (TEM)

Electron micrographs of the NE formulations were obtained using a JEOL GEM-1010 transmission electron microscope (JEOL Ltd., Tokyo, Japan) functioning at a voltage of 80 kV, with point-to-point resolution capabilities. In order to conduct the TEM analysis, a drop of the NE was placed onto carbon-coated copper grids (CCGs) and allowed to dry at ambient temperature (25 ± 2 °C), facilitating water evaporation and film formation prior to imaging [64]. The grids were examined at magnifications ranging from 30,000× to 100,000×, and representative micrographs were recorded. Each image was provided with a scale bar (typically 200 nm) to enable accurate interpretation of droplet size. These standardized imaging conditions ensured reproducibility and confirmed the nanoscale dimensions and spherical morphology of the formulations.

##### Refractive Index and Percentage Transmittance

The refractive index of each NE formulation was determined at 25 ± 0.5 °C using a Bellingham + Stanley RFM900-T refractometer (Bellingham + Stanley Ltd., Tunbridge Wells, UK). A single drop of each formulation was applied on the refractometer slide, and the readings were compared against the standard refractive index of water (1.333) to evaluate optical uniformity. Transparency was evaluated based on percentage transmittance using a UV-Visible spectrophotometer (UV-1700, Shimadzu Corporation, Kyoto, Japan) at a wavelength of 650 nm, with distilled water as blank reference.

##### Determination of Viscosity and pH

The viscosity of each NE formulation was measured using a cone and plate rheometer (Anton Paar, Graz, Austria) equipped with spindle CPE40. A sample volume of 0.5 g was analyzed at a temperature of 25 ± 0.5 °C. Data acquisition and analysis were performed using Rheocompass software (version 1.3). The pH of the NE formulations was determined using a calibrated digital pH meter (Mettler Toledo MP 220, Greifensee, Switzerland) at 25 °C. The measurements were conducted in triplicate to ensure reproducibility and accuracy.

#### 3.2.6. RP HPLC

A simple reverse-phase HPLC method was developed and validated for the quantification of APM in both bulk drug and NE formulations. The analysis was carried out using a Waters 1525 pump coupled with a Waters 2487 UV–VIS detector (Milford, MA, USA), and data acquisition was managed through Empower Pro software (Empower 3.10, Waters Corporation, Milford, MA, USA). Chromatographic separation was achieved on a Kromasil C18 column (250 mm × 4.6 mm, 5 µm). The mobile phase consisted of water and acetonitrile (30:70, *v*/*v*) with the addition of 0.1 mL triethanolamine, and the mixture was filtered through a 0.45 µm membrane filter (Millipore Corp., Madrid, Spain). The system was operated isocratically at 25 °C with a flow rate of 1 mL/min, an injection volume of 20 µL, and detection at 230 nm. Drug concentrations were determined by comparing the peak areas of test samples with those of standards of known concentration.

#### 3.2.7. Entrapment Efficiency

The entrapment efficiency of APM within the NE formulations was assessed via centrifugation. Formulations were centrifuged to separate the unencapsulated drug, and the resulting supernatant containing free APM was collected. Quantification of the free drug was carried out using a validated HPLC method at a detection wavelength of 230 nm, as described by Shofia et al. (2018) [65].

#### 3.2.8. Drug–Excipient Interaction Studies

FTIR analysis was performed to evaluate potential interactions between APM and excipients within the optimized NE formulation (F5). Spectra of pure APM and the selected formulation were recorded using a Nicolet iS50 FT-IR spectrometer (Thermo Fisher Scientific, Madison, WI, USA). Samples were scanned over a spectral range of 4000–400 cm^−1^ for the identification of characteristic functional groups and for assessing any shifts or changes indicative of molecular interactions [66].

#### 3.2.9. In Vitro Release and Release Kinetics Studies

Six NE formulations (F1–F6), each containing 2.5 mg/mL (0.25% *w*/*v*) APM, were evaluated for drug release using a Franz diffusion cell system (diffusion area: 1.77 cm^2^; receptor volume: 12 mL). A cellophane membrane, pre-soaked overnight in phosphate-buffered saline (PBS), was positioned between the donor and receptor chambers. The receptor compartment was filled with a PBS: methanol mixture (8:2 *v*/*v*) adjusted to pH 5.5, serving as the release medium. Formulations were placed in the donor chamber, and the system was kept at 37 ± 0.2 °C with constant stirring at 50 rpm [67]. Aliquots were collected from the receptor compartment at predetermined time intervals, and replaced with an equal volume of fresh release medium to preserve sink conditions. A control formulation comprising CR-APM (0.25% *w*/*v*) dissolved in the same oil phase was used for comparative analysis. Drug concentrations in the collected samples were quantified using a validated HPLC method at a detection wavelength of 230 nm. The release data were applied to multiple kinetic models, and the model showing the highest correlation coefficient (r^2^) was selected as the best-fit model.

Zero-order


(1) $$Q_{t}=k_{0}\cdot t+Q_{\infty }$$

First-order


(2) $$Q_{t}=Q_{\infty }(1-e^{-K_{f}\cdot t})$$

Higuchi equation


(3) $$Q_{t}=k_{H}\cdot t^{\frac{1}{2}}$$

#### 3.2.10. Preparation of APM-Loaded-NEG and CR-APMG

The NE formulation exhibiting the maximum drug release (F5) was selected as the optimized candidate for incorporation into a Carbopol-based gel (2% *w*/*w*). A pre-weighed quantity of Carbopol 980 polymer was dispersed in deionized water and homogenized vigorously to ensure uniform mixing. Triethanolamine (2–3 drops) was added gradually under continuous, gentle stirring to facilitate cross-linking and adjust the final pH to 7.0. The gel was left to stand overnight in a dark environment to eliminate entrapped air and complete polymer swelling. Subsequently, the optimized NE (F5) and a control formulation comprising CR-APMG, 0.25% *w*/*v* were dispersed in the Carbopol gel base in a 1:1 *w*/*w* ratio. Mixing was performed with gentle stirring until homogeneous gels were obtained [68].

#### 3.2.11. Evaluation of APM-NE Gel

##### pH and Viscosity

The pH of the NEG formulation (F5G) and CR-APMG was determined using a digital pH meter (Mettler Toledo MP 220, Greifensee, Switzerland). Calibration was performed using reference buffer solutions at pH 4.0, 7.0, and 9.0. For analysis, 1 g of the NEG formulation (F5G) and CR-APMG were dispersed in deionized water, stirred to achieve homogeneity, and kept to stand for 2 h. The volume of the dispersion was adjusted to 100 mL, and pH measurements were recorded in triplicate [69].

Viscosity of the APM-loaded-NEG (F5G) and the CR-APMG was measured using a cone and plate rheometer (Anton Paar, Graz, Austria) equipped with a CPE40 spindle. The analyses were performed at a temperature of 25 ± 0.5 °C, and data acquisition was facilitated using Rheocompass software (version 1.3).

##### Spreadability

The spreadability of the optimized NEG (F5G) and the CR-APMG was evaluated by placing one gram of each gel within a one-centimeter diameter circle, pre-marked on the central area of the glass plate using a parallel plate method. A second glass plate was gently positioned over the sample, followed by the placement of a 500-g weight to facilitate spreading. After 5 min, the diameter of the circle after the gels had spread was measured in triplicate at 25 °C [70]. The spreadability percentage was calculated using the following equation:

$$\%\;Spreadability=\frac{A_{2}}{A_{1}}\times 100$$

where A_1_ denotes 1 cm and A_2_ is the area that was measured after spreading.

#### 3.2.12. Drug Content Determination

To ensure uniform distribution of APM within the gel formulations, samples were collected from multiple locations within the mixing vessel and analyzed for drug content. A precisely weighed quantity (1.0 g) of each formulation, optimized NEG (F5G) and CR-APMG, was dissolved in methanol and homogenized using a vortex mixer for 2 min [71]. The obtained solutions were filtered to remove any undissolved excipients and subsequently analyzed using a validated HPLC method at a detection wavelength of 230 nm.

#### 3.2.13. Ex Vivo and In Vivo Animal Studies

##### Animals and Ethical Approval

Twenty-four male Wistar rats (220 ± 10.5 g) were procured from the Animal Care Unit, College of Medicine, Zagazig University (Zagazig, Ash Sharqiyah, Egypt), to evaluate the anti-inflammatory activity of the optimized NEG formulation (F5G). The animals were kept under a typical laboratory condition with regular light/dark cycles at an ambient temperature of 22 ± 2 °C, with free access to a standard diet and water. All experimental procedures were performed following institutional ethical guidelines after receiving approval from the Institutional Animal Care and Use Committee of Zagazig University (ZU-IACUC), under approval number ZU-IACUC/3/F/65/2024, approval date 27 March 2024.

##### Ex Vivo Skin Permeation Studies and Flux Profile

Ex vivo permeation studies were conducted using a Franz diffusion cell system to evaluate the transdermal delivery of APM through excised full-thickness skin from male Wistar rats (220 ± 10.5 g). The skin was carefully prepared by removing hair with an electric razor and excising subcutaneous fat using a sterile surgical blade. The tissue was then cleaned with methanol, rinsed with distilled water, and subsequently inspected visually to confirm its structural integrity. A 3.0 × 3.0 cm^2^ skin section was affixed to the Franz diffusion cell (diffusion area: 1.77 cm^2^; receptor volume: 12 mL).

The receptor chamber was loaded with a phosphate buffer: methanol mixture (80:20 *v*/*v*, pH 5.5) and kept at 37 ± 0.5 °C under constant stirring at 100 rpm [72]. The test formulations, optimized NE (F5), NEG (F5G), and CR-APMG were applied to the epidermal side of the mounted skin. At predetermined intervals (0, 0.5, 1, 2, 4, 6, 8, 10, 12, 18, and 24 h), aliquots of 0.5 mL were withdrawn from the receptor chamber and immediately replaced with fresh dissolution medium to preserve sink conditions. The obtained samples were analyzed in triplicate using a validated HPLC method at a detection wavelength of 230 nm.

The transdermal flux (J) and permeability coefficient (K_p_) were calculated using the following equation:

(4) $$J=\frac{dQ}{dtA}$$

(5) $$K_{p}=\frac{J_{ss}}{C_{o}}$$

where J denotes flux (μgcmh^−1^), dQ/dt denotes the slope derived from a linear curve, A denotes the area of diffusion (cm^2^), K_p_ denotes permeability coefficient, and C_o_ denotes the initial drug concentration in the donor cell.

##### Skin Deposition Studies

After the ex vivo permeation study was completed, the Franz diffusion cells were disassembled, and the skin samples were retrieved. To ensure removal of any residual surface formulation, the skin was thoroughly rinsed multiple times with the diffusion medium. The cleaned tissue was then finely minced to facilitate efficient extraction of the drug deposited within the skin layers. Methanol was used as the extraction solvent, and the resulting mixture was filtered and centrifuged at 10,000 rpm for 15 min. The supernatant was appropriately diluted and analyzed for APM content using a validated HPLC method.

##### Anti-Inflammatory Study

The anti-inflammatory efficacy of the optimized NEG formulation (F5G) was evaluated against marketed diclofenac sodium gel (1% Voltaren emulgel) and CR-APMG through the rat paw edema model using carrageenan for induction of inflammation [73,74]. A single-dose parallel study design was employed, involving twenty-four male Wistar rats randomly chosen and divided into four groups (*n* = 6 per group). Acute inflammation was induced by injecting 0.1 mL of a 1% (*w*/*v*) carrageenan solution into the left hind paw of each rat.

Group I received placebo Carbopol gel, while Group II, Group III, and Group IV received F5G, diclofenac sodium gel (1%), and CR-APMG, respectively. Treatments were applied to the sub plantar region immediately following carrageenan administration. The rats paw volume was measured at 0, 1, 2, 3, 4, and 5 h post-injection using a digital plethysmometer (LE 7500, Pan Lab, Harvard Apparatus, Barcelona, Spain).

The percentage inhibition of left paw edema was calculated using the following equation:

$$\%\;Inhibition\;of\;paw\;edema=\frac{T_{0}-T_{t}}{T_{0}}\times 100$$

where *T_t_* represents the thickness of the left paw in rats treated with the test sample at the specified time, while *T*_0_ indicates the paw thickness of rats in the carrageenan control group at that time.

##### Histopathological Studies

Following completion of the rat paw edema study, all animals were euthanized under anesthesia, and the left hind paw tissues were excised and preserved in 10% neutral buffered formalin. The preserved tissues were sliced at a thickness of 6 µm using a microtome and subsequently stained with eosin and haematoxylin (E&H) for histological examination. Tissue sections from all experimental groups—Group I (placebo gel), Group II (F5G), Group III (marketed diclofenac sodium gel), and Group IV (CR-APMG) were microscopically evaluated for signs of inflammation, including edema, neutrophil infiltration, and leukocyte accumulation. These findings were compared against histological profiles of the contralateral right paw tissues, which served as the normal control.

#### 3.2.14. In Vitro Anti-Inflammatory Studies

##### Cell Culture Conditions

The RAW 264.7 murine macrophage cell line was sourced from Nawah Scientific Inc. (Mokattam, Cairo, Egypt) and used for in vitro experiments. Cells were cultured in Dulbecco’s Modified Eagle Medium (DMEM; Biowest L0060 enriched with 10% heat-inactivated fetal bovine serum (FBS), 100 µg/mL streptomycin, and 100 U/mL penicillin). The cultures were incubated at 37 °C in a humidified atmosphere with 5% CO_2_ for 24 h, until the cells reached confluence [75].

##### Cell Viability Assay Using the Sulforhodamine B (SRB) Method

Cell viability was determined using the sulforhodamine B (SRB) assay. RAW 264.7 macrophages were seeded into 96-well plates at a density of 5 × 10^3^ cells/mL (100 µL per well) and incubated for 24 h at 37 °C in fully supplemented growth medium. The cells were subsequently treated with 100 µL of fresh medium containing varying concentrations (0.01, 0.1, 1, 10, and 100 µg/mL) of APM-NE and APM-loaded-NEG. Quercetin (30 µM) was included as a positive control. Following treatment, the cells were incubated for an additional 72 h under standard culture conditions.

To fix the cells, the medium was replaced with 150 µL of 10% trichloroacetic acid (TCA), followed by incubation at 4 °C for 1 h. The TCA solution was carefully removed, and wells were washed three to four times with distilled water. The fixed cells were stained with 70 µL of 0.4% (*w*/*v*) SRB solution and incubated in the dark at room temperature for 10 min. Excess dye was removed by rinsing the wells 3–4 times with 1% acetic acid, and the wells were left to air-dry overnight.

Finally, the protein-bound SRB dye was dissolved by adding 150 µL of 10 mM TRIS buffer to each well. Absorbance was measured at 540 nm using a FLUOstar Omega microplate reader (BMG LABTECH, Ortenberg, Germany). Absorbance was measured at 540 nm using a FLUOstar Omega microplate reader (BMG LABTECH, Ortenberg, Germany) operated with Reader Control Software V6.30 and MARS Data Analysis Software V5.02.

##### In Vitro Anti-Inflammatory Study

The anti-inflammatory activity of the APM-NE was investigated using RAW 264.7 murine macrophages. Cells were seeded into 96-well plates and maintained under standard culture conditions for 24 h. Inflammation was induced by treating the cells with lipopolysaccharide (LPS) at a concentration of 1 µg/mL (LPS group). In the control group, the culture medium was replaced with fresh medium without LPS. To evaluate the anti-inflammatory effect, LPS-stimulated cells were treated with APM-NE at different concentrations (LPS + drug group). Quercetin (30 µM) was used as a positive control.

Nitric oxide (NO) production was determined by mixing equal volumes of the cell culture supernatant and Griess reagent, followed by incubation in the dark at room temperature for 10 min. The concentration of nitrite, as an indicator of NO production, was measured spectrophotometrically at 540 nm using an ELISA plate reader [76].

### 3.3. Statistical Analysis

Statistical analyses were carried out using GraphPad Prism software (version 5.0; GraphPad Software Inc., San Diego, CA, USA). Results are expressed as mean ± standard deviation (SD). Differences between groups were evaluated by one-way analysis of variance (ANOVA), followed by Tukey’s post hoc multiple-comparison test. Statistical significance was defined as *p* < 0.05.

## 4. Conclusions

This study successfully developed and optimized APM-NE and its corresponding NEG formulation for the topical treatment of psoriasis. Among the various formulations, F5 comprising 18% Capryol 90, 50% Smix (Tween 20: Labrasol at 3:1), and 32% water exhibited superior physicochemical properties, including a nanoscale globule size (147.4 nm), low PDI (0.447), and zeta potential (−2.75 mV). The F5 formulation also demonstrated an optimal in vitro drug release profile (86.1 ± 0.24%).

To enhance skin retention and therapeutic efficacy, F5 was loaded into a Carbopol 980-based gel matrix to formulate F5G. Both F5 and F5G underwent comprehensive evaluation, including ex vivo skin permeation, in vitro anti-inflammatory testing using RAW 264.7 macrophages, and in vivo rat paw edema studies. The results revealed significantly improved drug deposition, enhanced anti-inflammatory activity, and superior skin permeation compared to CR-APMG and marketed diclofenac gel.

Overall, the APM-loaded-NEG formulation (F5G) demonstrated promising potential as a targeted and effective topical delivery system for the treatment of psoriasis, offering improved drug localization, sustained release, and enhanced therapeutic outcomes.

## Figures and Tables

**Table 1:** Physicochemical properties of apremilast-loaded nanoemulsions (formulations F1–F6).

NEs Code	PS * (nm)	PDI *	ZP (mV) *	RI *	%T *
F1	275.5 ± 8.2	0.623 ± 0.005	−2.87 ± 0.21	1.361 ± 0.02	96.94 ± 0.48
F2	231.6 ± 7.4	0.561 ± 0.003	−2.03 ± 0.18	1.341 ± 0.01	97.12 ± 0.18
F3	217.0 ± 6.6	0.522 ± 0.004	−2.66 ± 0.12	1.339 ± 0.06	97.62 ± 0.20
F4	195.06 ± 5.4	0.483 ± 0.012	−2.17 ± 0.24	1.344 ± 0.03	98.92 ± 0.28
F5	147.4 ± 2.4	0.447 ± 0.008	−2.75 ± 0.22	1.338 ± 0.04	99.57 ± 0.33
F6	165.9 ± 5.1	0.468 ± 0.013	−2.37 ± 0.28	1.349 ± 0.02	98.32 ± 0.46

* Mean ± standard deviation (*n* = 3). Abbreviations: NEs, nanoemulsions; PS, particle size; PDI, polydispersity index; ZP, zeta potential; RI, refractive index; T, transmission.

**Table 2:** Physicochemical characteristics of NE (F5), NEG (F5G), and CR-APMG.

Formulation Code	pH	Viscosity (mPa.s)	Spreadability		Drug Content (%)
			Diameter (cm)	Spreadability (%)	
F5	6.3 ± 0.03	54.1 ± 0.85 ***	–	–	99.15 ± 1.28
F5G	6.7 ± 0.04	730.1 ± 10.36 ***	6.5 ± 0.05 ***	650	97.82 ± 1.42
CR-APMG	7.2 ± 0.03	13,059 ± 41.25	4.1 ± 0.05	410	96.8 ± 2.37

Values are expressed as mean ± standard deviation (*n* = 3). Statistical differences among groups were analyzed by one-way ANOVA followed by Tukey’s post hoc test. *** *p* < 0.001. Abbreviations: F5, optimized apremilast nanoemulsion formulation; F5G, nanoemulsion gel derived from F5; CR-APMG, crude apremilast gel; mPa.s, millipascal-second.

**Table 3:** Permeation characteristics of F5, F5G, and CR-APMG across excised rat skin.

Formulation Code	Flux Jss (µg/cm^2^/h)	Permeability Coefficient Kp (×10^−3^ cm/h)	Enhancement Ratio ER
F5	28.90 ± 0.83 *	14.45 ± 0.002	2.02 ± 0.05
F5G	24.31 ± 0.22 *	9.72 ± 0.001	1.70 ± 0.02
CR-APMG	14.28 ± 0.18	5.71 ± 0.01	1.00

Values are expressed as mean ± standard deviation (*n* = 3). Statistical differences among groups were analyzed by one-way ANOVA followed by Tukey’s post hoc test. * *p* < 0.05. Abbreviations: Jss, steady state drug flux; F5, optimized apremilast nanoemulsion formulation; F5G, optimized apremilast nanoemulsion gel; CR-APMG, crude apremilast gel; ANOVA, analysis of variance.

**Table 4:** Composition of apremilast-loaded nanoemulsions (APM-NEs).

APM-NEs Codes	Components (% *v*/*v*)			S_mix_ Ratio
	Oil (Capryol 90)	S_mix_ (Tween 20 and Labrasol)	Water	
F1	7	33	60	1:1
F2	9	35	56	1:1
F3	12	40	48	2:1
F4	13	42	45	2:1
F5	18	50	32	3:1
F6	15	45	40	3:1

Abbreviations: F1–F6, apremilast nanoemulsion formulations prepared with varying compositions; S_mix_, surfactant/co-surfactant mixture.

## Data Availability

Data are contained within the article.

## References

[1] Parisi R., Symmons D.P.M., Griffiths C.E.M., Ashcroft D.M. (2013). The Identification and Management of Psoriasis and Associated Comorbidity project team. Global epidemiology of psoriasis: A systematic review of incidence and prevalence. J. Investig. Dermatol..

[2] Adışen E. (2022). Interleukin-23 inhibitors. Turkderm-Turk. Arch. Dermatol. Venereol..

[3] Raychaudhuri S.K., Maverakis E., Raychaudhuri S.P. (2014). Diagnosis and classification of psoriasis. Autoimmun. Rev..

[4] Kuchekar A., Pujari R., Kuchekar S., Dhole S., Mule P. (2011). Psoriasis: A comprehensive review. Int. J. Pharm. Life Sci..

[5] Lebwohl M., Sherer D., Washenik K., Krueger G.G., Menter A., Koo J., Feldman S.R. (2002). A randomized, double-blind, placebo-controlled study of clobetasol propionate 0.05% foam in the treatment of nonscalp psoriasis. Int. J. Dermatol..

[6] Barrea L., Savanelli M.C., Di Somma C., Napolitano M., Megna M., Colao A., Savastano S. (2017). Vitamin D and its role in psoriasis: An overview of the dermatologist and nutritionist. Rev. Endocr. Metab. Disord..

[7] Silva-Abreu M., Sosa L., Espinoza L.C., Fábrega M.-J., Rodríguez-Lagunas M.J., Mallandrich M., Calpena A.C., Garduño-Ramírez M.L., Rincón M. (2023). Efficacy of Apremilast Gels in Mouse Model of Imiquimod-Induced Psoriasis Skin Inflammation. Pharmaceutics.

[8] Wang C., Lin A. (2014). Efficacy of topical calcineurin inhibitors in psoriasis. J. Cutan. Med. Surg..

[9] Van De Kerkhof P.C. (2006). Update on retinoid therapy of psoriasis in: An update on the use of retinoids in dermatology. Dermatol. Ther..

[10] Zeichner J.A. (2010). Use of topical coal tar foam for the treatment of psoriasis in difficult-to-treat areas. J. Clin. Aesthet. Dermatol..

[11] Sehgal V.N., Verma P., Khurana A. (2014). Anthralin/dithranol in dermatology. Int. J. Dermatol..

[12] Dogra S., Mahajan R. (2013). Systemic methotrexate therapy for psoriasis: Past, present and future. Clin. Exp. Dermatol..

[13] Gyulai R., Bagot M., Griffiths C.E., Luger T., Naldi L., Paul C., Puig L., Kemény L., Psoriasis International Network (2015). Current practice of methotrexate use for psoriasis: Results of a worldwide survey among dermatologists. J. Eur. Acad. Dermatol. Venereol..

[14] Maza A., Montaudié H., Sbidian E., Gallini A., Aractingi S., Aubin F., Bachelez H., Cribier B., Joly P., Jullien D. (2011). Oral cyclosporin in psoriasis: A systematic review on treatment modalities, risk of kidney toxicity and evidence for use in non-plaque psoriasis. J. Eur. Acad. Dermatol. Venereol..

[15] Lee C.S., Koo J. (2005). A review of acitretin, a systemic retinoid for the treatment of psoriasis. Expert Opin. Pharmacother..

[16] Zhang P., Wu M.X. (2018). A clinical review of phototherapy for psoriasis. Lasers Med. Sci..

[17] Hire P., Gondkar S., Bachhav R. (2025). Formulation development and evaluatio Formulation, Optimization and in vitro Evaluation of Apremilast Nanoemulgel for Topical Delivery n of topical nanoemulgel of apremilast. World J. Pharm. Med. Res..

[18] Kitzen J., Pergolizzi J., Taylor R., Raffa R. (2018). Crisaborole and Apremilast: PDE4 Inhibitors with Similar Mechanism of Action, Different Indications for Management of Inflammatory Skin Conditions. Pharmacol. Pharm..

[19] Li H., Zuo J., Tang W. (2018). Phosphodiesterase-4 Inhibitors for the Treatment of Inflammatory Diseases. Front. Pharmacol..

[20] Mulleria S.S., Marina K., Ghetia S.M. (2021). Formulation, Optimization and in vitro Evaluation of Apremilast Nanoemulgel for Topical Delivery. Int. J. Pharm. Investig..

[21] Schett G., Wollenhaupt J., Papp K., Joos R., Rodrigues J.F., Vessey A.R., Hu A., Stevens R., de Vlam K.L. (2012). Oral apremilast in the treatment of active psoriatic arthritis: Results of a multicenter, randomized, double-blind, placebo-controlled study. Arthritis Rheum..

[22] Sharma V., Jami V., Setti M.L.V., Choudhury A.A., Basha A.M. (2022). Optimization, evaluation and comparative IVPT study of micro and nano liposomal topical formulations of apremilast. Mater. Today Proc..

[23] Parmar P.K., Bansal A.K. (2021). Novel nanocrystal-based formulations of apremilast for improved topical delivery. Drug Deliv. Transl. Res..

[24] Yang L., Wu P., Xu J., Xie D., Wang Z., Wang Q., Chen Y., Li C.H., Zhang J., Chen H. (2021). Development of apremilast solid dispersion using TPGS and PVPVA with enhanced solubility and bioavailability. AAPS PharmSciTech.

[25] Shetty D., Yarlagadda D.L., Brahmam B., Dengale S.J., Lewis S.A. (2023). Investigating the influence of the type of polymer on sustaining the supersaturation from amorphous solid dispersions of Apremilast and its pharmacokinetics. J. Drug Deliv. Sci. Technol..

[26] Alfehaid F.S., Nair A.B., Shah H., Aldhubiab B., Shah J., Mewada V., Jacob S., Attimarad M. (2024). Enhanced transdermal delivery of apremilast loaded ethosomes: Optimization, characterization and in vivo evaluation. J. Drug Deliv. Sci. Technol..

[27] Rahangdale M., Pandey P. (2021). Development and characterization of apremilast transethosomal gel for transdermal delivery. Int. J. Pharm. Sci. Nanotechnol..

[28] Rapalli V.K., Sharma S., Roy A., Alexander A., Singhvi G. (2021). Solid lipid nanocarriers embedded hydrogel for topical delivery of apremilast: In-vitro, ex-vivo, dermatopharmacokinetic and anti-psoriatic evaluation. J. Drug Deliv. Sci. Technol..

[29] Ahmed M.M., Anwer M.K., Fatima F., Alali A.S., Kalam M.A., Zafar A., Alshehri S., Ghoneim M.M. (2022). Development of apremilast nanoemulsion-loaded chitosan gels: In vitro evaluations and anti-inflammatory and wound healing studies on a rat model. Gels.

[30] Zhao L., Chen J., Bai B., Song G., Zhang J., Yu H., Huang S., Wang Z., Lu G. (2024). Topical drug delivery strategies for enhancing drug effectiveness by skin barriers and delivery systems. Front. Pharmacol..

[31] Shakeel F., Raish M., Anwer M.K., Al-Shdefat R.I. (2016). Self-nanoemulsifying drug delivery system of sinapic acid: In vitro and in vivo evaluation. J. Mol. Liq..

[32] Elsewedy H.S. (2025). Insights of Nanoemulsion as a Drug Delivery System: An Overview of Current Trends and Applications. Indian J. Pharm. Educ. Res..

[33] Maestrelli F., Gonzalez-Rodriguez M.L., Rabasco A.M., Mura P. (2006). Effect of preparation technique on the properties of liposomes encapsulating ketoprofen-cyclodextrin complexes aimed for transdermal delivery. Int. J. Pharm..

[34] Naz Z., Ahmad F.J. (2015). Curcumin-loaded colloidal carrier system: Formulation optimization, mechanistic insight, ex vivo and in vivo evaluation. Int. J. Nanomed..

[35] Akhter A., Shirazi J.H., Shoaib Khan H.M., Hussain M.D., Kazi M. (2024). Development and evaluation of nanoemulsion gel loaded with bioactive extract of Cucumis melo var. agrestis: A novel approach for enhanced skin permeability and antifungal activity. Heliyon.

[36] Ali A., Ansari V.A., Ahmad U., Akhtar J., Jahan A. (2017). Nanoemulsion: An Advanced Vehicle for Efficient Drug Delivery. Drug Res..

[37] Li P., Ghosh A., Wagner R.F., Joshi Y.M., Serajuddin A.T.M. (2005). Effect of combined use of nonionic surfactant on formation of oil-in-water microemulsions. Int. J. Pharm..

[38] Suma R., Shailesh K., Chakraborty T., Khanum A., Kumar S.H. (2025). Design and development of microemulsion of apremilast as a potential doasge form for the efficient treatment of psoriatic nail dystrophy through transungual route. Int. J. Pharm. Sci. Res..

[39] Suryawanshi R.M., Gilhotra R.M., Dhakad P.K., Gupta T. (2025). Formulation Design and Development of Azilsartan Nano-emulsion for the Solubility Enhancement. Int. J. Drug Deliv. Technol..

[40] Juniatik M., Hidayati K., Wulandari F.P., Pangestuti N., Munawaroh N., Martien R., Utami S. (2017). Formulation of nanoemulsion mouthwash combination of lemongrass oil (*Cymbopogon citratus*) and kaffir lime oil (*Citrus hystrix*) against *Candida albicans* ATCC 10231. Tradit. Med. J..

[41] Kumar S. (2014). Role of nano-emulsion in pharmaceutical sciences—A review. AJRPSB.

[42] Danaei M., Dehghankhold M., Ataei S., Hasanzadeh Davarani F., Javanmard R., Dokhani A., Khorasani S., Mozafari M.R. (2018). Impact of Particle Size and Polydispersity Index on the Clinical Applications of Lipidic Nanocarrier Systems. Pharmaceutics.

[43] Jores K., Mehnert W., Drechsler M., Bunjes H., Johann C., Mäder K. (2004). Investigations on the structure of solid lipid nanoparticles by atomic force microscopy. Int. J. Pharm..

[44] Warisnoicharoen W., Lansley A.B., Lawrence M.J. (2000). Nonionic oil-in-water microemulsions: The effect of oil type on phase behaviour. Int. J. Pharm..

[45] Oliveira C.A., Gouvea M.M., Antunes G.R., de Freitas Z.M.F., de Carvalho Marques F.F., Ricci Junior E. (2018). Nanoemulsion containing 8-methoxypsoralen for topical treatment of dermatoses: Development, characterization and ex vivo permeation in porcine skin. Int. J. Pharm..

[46] Malik M.R., Al-Harbi F.F., Nawaz A., Amin A., Farid A., Mohaini M.A., Alsalman A.J., Hawaj M.A.A., Alhashem Y.N. (2022). Formulation and Characterization of Chitosan Decorated Multiple Nanoemulsion for Topical Delivery in vitro and ex vivo. Molecules.

[47] Panonnummal R., Jayakumar R., Sabitha M. (2017). Comparative anti-psoriatic efficacy studies of clobetasol loaded chitin nanogel and marketed cream. Eur. J. Pharm. Sci..

[48] Xiong K., Ma X., Cao N., Liu L., Sun L., Zou Q., Wei P. (2016). Identification, characterization and HPLC quantification of impurities in apremilast. Anal. Methods.

[49] Rai V.K., Mishra N., Yadav K.S., Yadav N.P. (2018). Nanoemulsion as pharmaceutical carrier for dermal and transdermal drug delivery: Formulation development, stability issues, basic considerations and applications. J. Control. Release.

[50] Zahid F., Batool S., Ud-Din F., Ali Z., Nabi M., Khan S., Salman O., Khan G.M. (2022). Antileishmanial Agents Co-loaded in Transfersomes with Enhanced Macrophage Uptake and Reduced Toxicity. AAPS PharmSciTech.

[51] Nurman S., Yulia R., Irmayanti, Noor E., Sunarti T.C. (2019). The Optimization of Gel Preparations Using the Active Compounds of Arabica Coffee Ground Nanoparticles. Sci. Pharm..

[52] Sharma S., Sahni J.K., Ali J., Baboota S. (2015). Effect of high pressure homogenization on formulation of TPGS loaded nanoemulsion of rutin—Pharmacodynamic and antioxidant studies. Drug Deliv..

[53] Zhang X., Retyunskiy V., Qiao S., Zhao Y., Tzeng C.-M. (2022). Alloferon-1 ameliorates acute inflammatory responses in λ-carrageenan-induced paw edema in mice. Sci. Rep..

[54] Elsamman M., El-Borady O.M., Nasr M.M., Al-Amgad Z., Metwally A.A. (2024). Development of propolis, hyaluronic acid, and vitamin K nano-emulsion for the treatment of second-degree burns in albino rats. BMC Complement. Med. Ther..

[55] Zhang G.B., He Z.F., Shang W.Z., Wang A.W., Wu Y.F., Huang H.Y. (2022). Effect of triethanolamine on the biological characteristics of diffuse large B-cell lymphoma cells. Chin. J. Gen. Pract..

[56] Kaštelan M., Prpić-Massari L., Brajac I. (2009). Apoptosis in psoriasis. Acta Dermatovenerol. Croat..

[57] Lowes M.A., Russell C.B., Martin D.A., Towne J.E., Krueger J.G. (2013). The IL-23/T17 pathogenic axis in psoriasis is amplified by keratinocyte responses. Trends Immunol..

[58] Spiclin P., Homar M., Zupancic V.A., Gasperlin M. (2003). Sodium ascorbyl phosphate in topical microemulsions. Int. J. Pharm..

[59] Shafiq-un-Nabi S., Shakeel F., Talegaonkar S., Ali J., Baboota S., Ahuja A., Khar R.K., Ali M. (2007). Formulation development and optimization using nanoemulsion technique: A technical note. AAPS PharmSciTech.

[60] Shakeel F., Haq N., Alanazi F.K., Alsarra I. (2013). Impact of various nonionic surfactants on self-nanoemulsification efficiency of two grades of Capryol (Capryol-90 and Capryol-PGMC). J. Mol. Liq..

[61] Abushal A.S., Aleanizy F.S., Alqahtani F.Y., Shakeel F., Iqbal M., Haq N., Alsarra I.A. (2022). Self-Nanoemulsifying Drug Delivery System (SNEDDS) of Apremilast: In Vitro Evaluation and Pharmacokinetics Studies. Molecules.

[62] Sureshkumar R., Gowthamarajan K., Bhavani P. (2015). Nanoemulsion for lymphatic absorption: Investigation of fenofibrate nanoemulsion system for lymphatic uptake. Int. J. Chem. Tech. Res..

[63] Sarango-Granda P., Silva-Abreu M., Calpena A., Halbaut L., Fábrega M.J., Rodríguez-Lagunas M., Díaz-Garrido N., Badia J., Espinoza L. (2020). Apremilast Microemulsion as Topical Therapy for Local Inflammation: Design, Characterization and Efficacy Evaluation. Pharmaceuticals.

[64] Amin B.H., Ahmed H.Y., El Gazzar E.M., Badawy M.M.M. (2021). Enhancement the Mycosynthesis of Selenium Nanoparticles by Using Gamma Radiation. Dose Response.

[65] Shofia S.I., Jayakumar K., Mukherjee A., Chandrasekaran N. (2018). Efficiency of brown seaweed (*Sargassum longifolium*) polysaccharides encapsulated in nanoemulsion and nanostructured lipid carrier against colon cancer cell lines HCT 116. RSC Adv..

[66] Khuroo T., Mohamed E.M., Dharani S., Immadi S., Nutan M.T., Lu D., Ali H.I., Khan M.A., Rahman Z. (2022). In-situ implant formulation of laurate and myristate prodrugs of dolutegravir for ultra-long delivery. J. Pharm. Sci..

[67] Kajbafvala A., Salabat A., Salimi A. (2018). Formulation, characterization, and in vitro/ex vivo evaluation of quercetin-loaded microemulsion for topical application. Pharm. Dev. Technol..

[68] Jeengar M.K., Rompicharla S.V., Shrivastava S., Chella N., Shastri N.R., Naidu V.G., Sistla R. (2016). Emu oil based nano-emulgel for topical delivery of curcumin. Int. J. Pharm..

[69] Ganarajan G., Sharma D.C., Tangri P., Kothiyal P. (2018). Design and characterization of apremilast loaded emulgel for topical treatment. Int. J. Pharm. Biol. Sci..

[70] Coneac G.H., Vlaia V., Olariu I.V., Muț A.M., Anghel D.F., Ilie C.I., Popoiu C.M., Lupuleasa D., Vlaia L.L. (2015). Development and Evaluation of New Microemulsion-Based Hydrogel Formulations for Topical Delivery of Fluconazole. AAPS PharmSciTech.

[71] Tas C., Ozkan Y., Savaser S., Baykara T. (2003). In vitro release studies of chlorpheniramine maleate from gels prepared by different cellulose derivatives. IL Farm..

[72] Madan J.R., Khobaragade S., Dua K., Awasthi R. (2020). Formulation, optimization, and in vitro evaluation of nanostructured lipid carriers for topical delivery of Apremilast. Dermatol. Ther..

[73] Ansari M.J., Alshetaili A., Aldayel I.A., Alablan F.M., Alsulays B., Alshahrani S., Alalaiwe A., Ansari M.N., Ur Rehman N., Shakeel F. (2020). Formulation, characterization, in vitro and in vivo evaluations of self-nanoemulsifying drug delivery system of luteolin. J. Taibah Univ. Sci..

[74] Faisal M.S., Eljroushi Z.M., Sawan M.S. (2021). Anti-inflammatory activity of ethanolic extract of Cnicus Benedictus. Lebda Med. J..

[75] Gharred N., Ali L.M.A., Bettache N., Dridi-Dhaouadi S., Morere A., Menut C. (2023). In Vitro Anti-inflammatory Activity of Three Inula Species Essential Oils in Lipopolysaccharide-Stimulated RAW 264.7 Macrophages. Chem. Afr..

[76] Kim C., Le D., Lee M. (2021). Diterpenoids Isolated from Podocarpus macrophyllus Inhibited the Inflammatory Mediators in LPS-Induced HT-29 and RAW 264.7 Cells. Molecules.

